# Deep Learning-Based NMPC for Local Motion Planning of Last-Mile Delivery Robot

**DOI:** 10.3390/s22218101

**Published:** 2022-10-22

**Authors:** Muhammad Imad, Oualid Doukhi, Deok Jin Lee, Ji chul Kim, Yeong Jae Kim

**Affiliations:** 1 Department of Mechanical Design Engineering, Jeonbuk National University, Jeonju-si 54896, Korea; 2 Department of Smart Machine Technology, Korea Institute of Machinery & Materials (KIMM), Daejeon 34103, Korea

**Keywords:** semantic segmentation, obstacle avoidance, zero-shot transfer, sidewalk autonomous delivery robots

## Abstract

Feasible local motion planning for autonomous mobile robots in dynamic environments requires predicting how the scene evolves. Conventional navigation stakes rely on a local map to represent how a dynamic scene changes over time. However, these navigation stakes depend highly on the accuracy of the environmental map and the number of obstacles. This study uses semantic segmentation-based drivable area estimation as an alternative representation to assist with local motion planning. Notably, a realistic 3D simulator based on an Unreal Engine was created to generate a synthetic dataset under different weather conditions. A transfer learning technique was used to train the encoder-decoder model to segment free space from the occupied sidewalk environment. The local planner uses a nonlinear model predictive control (NMPC) scheme that inputs the estimated drivable space, the state of the robot, and a global plan to produce safe velocity commands that minimize the tracking cost and actuator effort while avoiding collisions with dynamic and static obstacles. The proposed approach achieves zero-shot transfer from a simulation to real-world environments that have never been experienced during training. Several intensive experiments were conducted and compared with the dynamic window approach (DWA) to demonstrate the effectiveness of our system in dynamic sidewalk environments.

## 1. Introduction

The autonomous systems domain is developing extensively worldwide in terms of a variety of new utilities and the degree of interest among long-established players in the civilian and military communities [1,2]. Autonomous robotic systems have great potential for enhancing safety and real-time operational efficiency [3]. Furthermore, owing to the mercurial growth of the e-commerce industry, the need for efficient, affordable, and feasible last-mile deliveries has become more critical [4]. Sidewalk autonomous delivery robots (SADRs) are one of these potential technologies and have been the focus of research in recent years [5]. The SADRs were designed and engineered to deal with unexpected real-world scenarios without human intervention. Therefore, SADRs need to achieve their tasks in ambivalent and amorphous conditions, which pushes them to rely on their perception systems to perceive the surrounding environment while avoiding obstacles in real-time, which is also a performance indicator for mobile robots. Considering these factors, a mobile delivery robot with autonomous navigation potential has a higher application value, which is a vital area for the further expansion of mobile robot applications and is considered a critical factor in maximizing the intelligence level of the logistics industry.

The deployment of SADRs relies primarily on three major tasks: perception of the environment, path planning, and subsequent vehicle controls. Whereas the latter has proven to be a solved problem, the first two are still limited and have to be improved. Some of the suggested solutions involve the implementation of GPS devices accompanied by three-dimensional LiDARs and inertial measurement (IMU) [6,7], while a few utilize 2D LiDARs [8,9]. However, a wide range of implementation of these methods is quite difficult owing to the high cost, complexity, and unpredictability in specific scenarios. Existing methods have addressed these problems by dividing the robot navigation task into two modules: a global planning module, which creates trajectories from the robot’s current position to a given target goal [10], and a path-following module, which keeps the robot close to the planned path [11]. However, both modules depend on environmental characteristics and robot dynamics, making them sensitive and requiring re-tuning for each scheme. Moreover, these methods suffer from the stochastic dynamic behavior of obstacles and high computational costs when applied to highly unstructured environments.

More recently, researchers have proposed modular and end-to-end-based approaches that have proven effective in various vision-based robotic perception tasks [12,13]. A model-based cognitive mapping and planning approach was successfully illustrated in first-person visual navigation [14,15]. Reinforcement learning (RL) has been utilized for robot goal-driven navigation using visual input data [16]. Applying end-to-end supervised learning to steer a drone in addition to a manually labeled image-action pair was presented in [17]. An end-to-end training methodology for object manipulation tasks using unlabeled video data was described in [18]. Although these learning-based approaches seem captivating, they usually require an immense amount of training data. Piling up learnable data that should cover all types of scenarios for learning a control policy is costly and time-consuming and poses several challenges. Training a supervised learning-based model from scratch requires a large, diverse dataset and considerable human effort to label the data. However, RL depends on trial-and-error experiences, limiting the ability of dainty robots to perform various tasks. Moreover, online training in real-world scenarios is costly and prone to failure, thereby minimizing the learning capability of most RL algorithms.

A substitute for the aforementioned methods is to train the robot in a virtual environment to increase its capability and reduce training costs. Most methods based on this idea rely on gathering labeled data from a virtual environment [19,20,21]. However, the data collected from these virtual simulated environments have a considerable reality gap and are task-specific. For instance, to collect a sidewalk dataset for a customized delivery robot, it is impossible to use the available self-driving car simulators in a sidewalk environment because the data collected with a car configuration cannot be used to train a delivery robot. Therefore, defining a customized simulated environment, collecting data from that environment, training a model, and then transferring that model from the virtual to the real world have been challenging problems in computer vision and robotics.

**Figure 1:**
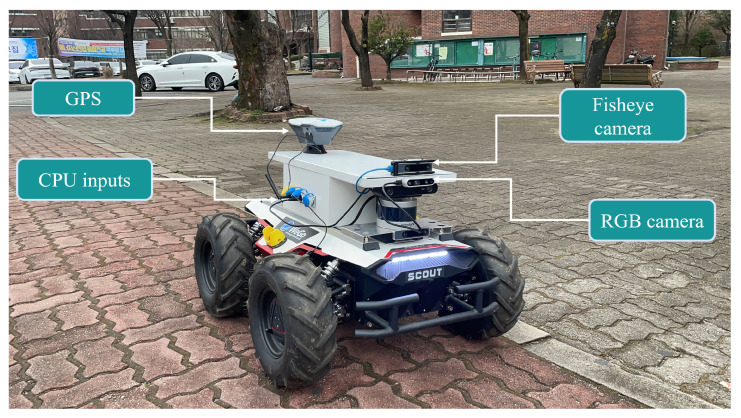
The customized delivery robot platform.

This paper presents a deep learning-based mapless local motion-planning technique for an autonomous mobile robot. First, a realistic simulator was developed based on Unreal Engine [22]. Second, a synthetic dataset was collected to train the deep learning module. The developed algorithm exploits semantic segmentation and a local motion planner property to provide low-level safe motion commands that drive SADRs (see Figure 1) on a sidewalk while avoiding dynamic and static obstacles. The proposed technique fuses information from the semantic segmentation module, GPS, and IMU to provide a robust motion plane in challenging situations without relying on mapping. A nonlinear model predictive (NMPC) [23] was used in the proposed method, which is considered a popular solution in local trajectory planning, owing to the advantages of movement fluency while considering the robot’s kinematic constraints, such as linear and angular speed limit.

However, existing traditional NMPC is prone to failure if there is no accurate obstacle information. Accordingly, a new NMPC cost function was proposed. It uses semantic segmentation to define the drivable area for the SADRs and enables the ability to perceive the obstacles in the surrounding environment, which allows the robot to avoid colliding with them. Computer simulations and real-world demonstrations show that the efficiency of the improved NMPC is increased in terms of motion safety and smoothness.

For training and experimentation, a dataset was collected from different simulated sidewalk scenarios, including harsh weather conditions, such as fog and snow. The dataset was labeled manually to train a semantic segmentation model. A transfer learning technique with a few frames from each scenario was used to increase the model generalization ability and achieve zero-shot sim-to-real transfer. The proposed architecture was separated into two modules: perception and local motion planner modules. The perception module converts RGB images into semantic segmentation using the trained model, which defines the drivable area. The local motion planner uses the obtained segmentation results to plan a set of trajectories within 6 seconds of prediction. A cost function was designed to select the most feasible trajectory that achieves obstacle avoidance and global path tracking. The perception module uses a convolution neural network-based encoder–decoder architecture, where MobileNetv2 [24] serves as an encoder, and on top of that, a simple yet efficient decoder has been defined to uplift low-level information. As the segmentation output generated by the synthetic and real data perception models is invariant, the model was deployed directly into the real world without further training and fine-tuning. The proposed architecture is more general and can be deployed in various scenarios and tasks. Unlike [25], which uses multiple-class segmentation, which increases the computational cost of the model, we use a binary segmentation mask to bridge the visual and planning modules. The mobile delivery robot is supposed to steer on sidewalks and streets and not on the main road. Therefore, we do not need to segment obstacles, such as cars, motorbikes, or trucks. The segmentation mask was divided into binary classes: drivable areas and obstacles, including people, light poles, and benches.

To demonstrate the validity of our algorithm, the proposed model and the DeeplabV3 [26], PSPNET [27], FCN [28], and U-Net [29] were trained on simulated data, and their performances were compared in benchmark tasks in both the virtual and real world. In addition, the local motion-planning module was compared to the dynamic window approach. The obtained results demonstrate the effectiveness of the proposed method in terms of obstacle avoidance, motion smoothness, and sim-to-real perception system transferability.

The remainder of this paper is organized as follows: Section II introduces related work. In Section III, the proposed modular architecture is discussed. Section IV describes the evaluation setup, tasks, and scenarios. Section V presents experimental results. Finally, Section VI concludes the proposed work.

## 2. Related Work

In recent years, vision-based autonomous navigation has become a popular field in applications ranging from intelligent vehicles to autonomous robots [25,30]. For instance, Zaidner [31], established a data fusion scheme for navigation, which effectively fused the localization data from multiple sensors; visual odometry (VO), GPS, inertial navigation system (INS), and wheel odometry. However, as highlighted by the author, there is a trade-off between cost and accuracy, and the data fusion algorithm can fail if the sensors significantly differ from each other. In [32], the autonomous navigation of mobile robots based on cognitive development was proposed, which achieves the expected navigation target by simulating animals, but the navigation path is not optimal. In [33], autonomous navigation based on fuzzy logic and reinforcement learning was investigated. Although the simulation results show that the method can significantly improve the behavior and learning speed of a robot, autonomous navigation cannot be implemented quickly. In [34], a mobile robot path planning method based on deep reinforcement learning was proposed. The learned policy selects the optimal mobile action that allows the mobile robot to reach the target position while avoiding the obstacles.

Several other studies have been proposed for autonomous and collision avoidance when obstacle locations and the global environment are known [35,36]. For instance, this scheme [37] presents a mobile robot navigation control system based on the integration of laser SLAM localization and real-time collision avoidance control to provide personnel guidance for daily life services. However, these techniques depend highly on the environmental map representation method (metric or feature-based). One of the predominant advantages of our scheme over these other methods is that it does not require a prior map of the surroundings. With the current developments of edge computing systems, alternative approaches have been proposed for reactive motion planning, where the system depends on the instant perception of its surrounding environment for decision-making.

The work in [38] proposed an end-to-end method for training deep learning models to infer the best action given an input color image. Another study [39] presented a data-driven motion planner based on a CNN model, which was trained using an existing motion planner. However, these studies are still limited in their generalization capabilities for new inexperienced scenarios. These issues have caused the research community to shift its focus to different technologies.

More recently, alternative approaches to robot motion planning have been based on semantic segmentation techniques. The work in [40] proposed a vision-based navigation system where in the first phase, a supervised generative network is trained to map outdoor images with rich information. Second, binary semantic segmentation was used to differentiate between obstacles and roads. Although the results verify the importance of the proposed method, running a two-stage perception module is computationally expensive and the proposed solution does not provide information on how binary segmentation is used in navigation.

Unlike the previous method, the authors of [41] exploited a custom-trained segmentation network and a low-RGB camera to produce smooth trajectories and stable control in different vineyard scenarios. Although the method showed outstanding results, this framework cannot be directly translated into an unstructured, dynamic environment, such as a sidewalk.

Other work, such as [42], proposed a vision-based navigation scheme that enafbles autonomous movement in indoor scenes; only a webcam was used as a perception sensor. However, as the name suggests, this method is restricted only to the indoor environment. A vision-based navigation scheme was presented in [43]. It uses a deep CNN to recognize road regions in front of the robot. Although the results demonstrate the possibility of visual navigation using only a single camera, the proposed method requires a large dataset based on real-world scenarios for training, which is time-consuming.

## 3. Proposed Methodology

Many conventional motion-planning methods can only handle static obstacles. The proposed algorithm does not require obstacles to be static for navigation. This section describes an autonomous navigation technique for mobile delivery robots. The objective is to retrieve an optimal trajectory $T^{*}$ given the estimated drivable area $d_{t}$ from the perception module.

### 3.1. Problem Formulation

**Figure 2:**
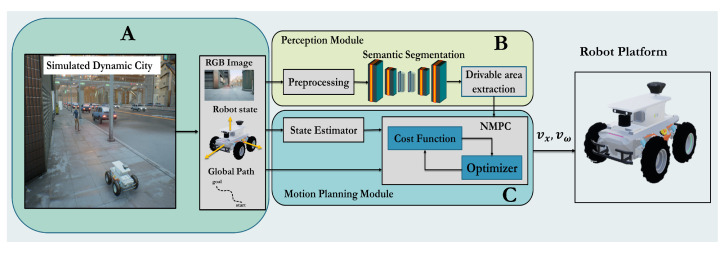
The overall block diagram of the proposed algorithms: (**A**) Unreal-Engine-based simulation of dynamic city with moving pedestrian. (**B**) The perception module based on semantic segmentation. (**C**) Local motion planner based on NMPC.

The proposed modular architecture contains a perception module (PM) and motion planner module (MPM) see Figure 2. PM is responsible for estimating the drivable area $d_{t}$. The MPM takes the drivable surface $d_{t}$, robot pose $P_{t}$, and global waypoint $w_{t}$ as inputs and returns the best trajectory $T^{*}$ that minimizes the optimization objective *J*, which ensures obstacle avoidance, motion smoothness, and reaching the global goal.

### 3.2. Perception Module

**Figure 3:**
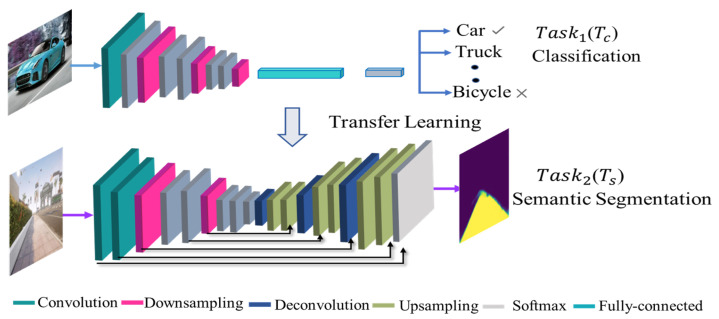
Training of a pre-trained model using transfer learning and testing in real-world scenarios.

The primary function of the perception module is to estimate the drivable area, $d_{t}$, and pass it to MPM. During the training stage, $d_{t}$ is learned from a synthetic image $x_{t}^{syn}$ generated from an Unreal-Engine-based simulator. Afterward, in the testing phase, the perception module uses real color images, $x_{t}^{real}$. Its function is expressed as:

(1) $$\begin{array}{c} d_{t}=S\left(x_{t}^{real},\Theta^{*}\right), \end{array}$$

where *S* represents the segmentation model, and $\Theta^{*}$ denotes the optimal model parameter. The proposed model *S* employed a transfer learning-based encoder–decoder architecture, as shown in Figure 3, where the classification model is transferred into a semantic segmentation model, which not only minimizes the need for a large dataset but also decreases the training time and increases the model accuracy.

MobileNetV2 was adopted as an encoder for model *S*, containing a convolutional layer combined with downsampling layers to produce a low-resolution tensor for high-level information. The architecture of the encoder is listed in Table 1. The decoder consists of matching convolutional layers coupled with upsampling layers to increase the size of the spatial tensor and to generate a high-resolution segmentation output. However, simply piling the encoder and the decoder architecture can result in a loss of low-level information. Therefore, the segmentation map boundaries initiated by the decoder will be faulty. Consequently, the decoder was allowed to access the low-level features produced by the encoder layers through skip connections. The intermediate outputs of the encoder are anchored to the inputs to the median layers of the decoder at the relevant position, as shown in Figure 3. Model *S* was designed to achieve the two main characteristics. First, although the visual appearances of $x_{t}^{real}$ and $x_{t}^{syn}$ differ, their semantic segmentation output is invariant, which implies that model *S* allows zero-shot sim-to-real transfer while being trained entirely using synthetic data. Second, segmentation model *S* only uses a monocular camera as input, which is cheaper than depth cameras or laser rangefinders. A synthetic dataset of 800 images with their corresponding binary mask was used for training. First, all the obtained images were reshaped to a fixed input dimension, 24×24, and normalized with the value from 0 to 1. Afterward, the model was trained for 50 epochs with a learning rate lr=0.0003 and a batch size of 4. The Nvidia GTX 1080 GPU with 16 GB of memory and Cuda 11 with Tensorflow 2.2 were utilized for training. With the mentioned hardware configuration, the training phase of the segmentation head takes approximately 30 min.

### 3.3. Local Motion Planning Module

Local motion planning is crucial for robots operating in unstructured dynamic environments. Feasible and safe trajectories that allow obstacle avoidance are crucial in such environments. The proposed MPM combines optimal motion planning and optimal control into a unified nonlinear model predictive control (NMPC) with constraints on states and manipulated variables, intending to minimize a customized cost function *J* that guarantees global waypoint tracking, actuator effort minimization, and obstacle avoidance by exploiting the segmented drivable area obtained from the perception module. At each iteration of the NMPC, the following optimization problem (see Equation (2)) was solved based on the latest robot state measurement, $x_{k}$ at time *k*. The first optimized control input *U* within the specified horizon is applied until a new NMPC update arrives.

(2a) $$\begin{array}{cc} \min_{U(.)}\; & \sum_{k=k_{s}}^{k_{f}}J\left(x\left(k\right),U\left(k\right),k\right), \end{array}$$

(2b) $$\begin{array}{cc} \mathrm{subject}\mathrm{to}\; & x\left(k_{s}\right)=x_{s}, \end{array}$$

(2c) $$\begin{array}{cc} \; & x(k+1)=x(k)+v(k)cos(\psi (k)), \end{array}$$

(2d) $$\begin{array}{cc} \; & y(k+1)=y(k)+v(k)sin(\psi (k)), \end{array}$$

(2e) $$\begin{array}{cc} \; & \psi \left(k+1\right)=\psi \left(k\right)+v_{\psi }\left(k\right), \end{array}$$

(2f) $$\begin{array}{cc} \; & v_{min}\leq U_{1}\leq v_{max}, \end{array}$$

(2g) $$\begin{array}{cc} \; & v_{\psi_{min}}\leq U_{2}\leq v_{\psi_{max}}, \end{array}$$

where x(k) is the state, $U\left(k\right)=\left[U_{1},U_{2}\right]$ is the linear and angular control input at time *t* and *J* is a time-varying cost function. The objective is to retrieve a control input that minimizes the cost (2a) subject to the robot’s initial state (2b), system kinematics (2c), (2d), (2e), and general inequality (2f) and (2g) constraints. As shown in Equation (3), the cost function *J* contains two parts, $J_{c}$ for obstacle avoidance and $J_{s}$ for waypoint tracking.

(3) $$J\left(x,k,U\right)=J_{c}\left(x,k\right)+J_{s}\left(x,k,U\right).$$

The $J_{s}\left(x,k,U\right)$ has a quadratic form with the goal of minimizing the distance between the robot and the given waypoint $w_{r}$, while considering the minimization of the actuator efforts, as presented in Equation (4).

(4) $$J_{s}\left(x,k,U\right)=\frac{1}{2}∥x-w_{r}{∥}^{2}Q+\frac{1}{2}{∥U-U_{r}∥}^{2}R,$$

where *R* and *Q* are the positive semi-definite weighting matrices. $U_{r}$ denotes the equilibrium control input vector.

**Figure 4:**
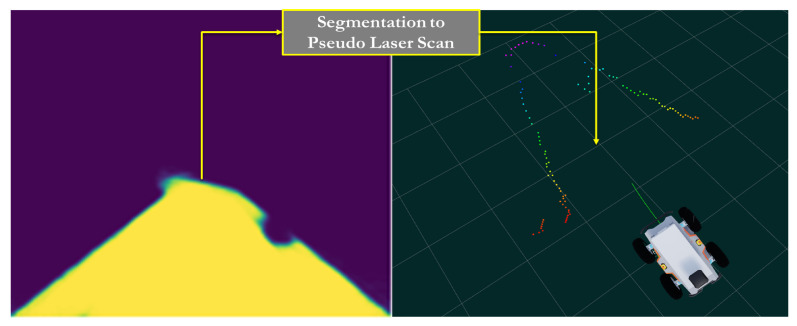
From semantic segmentation to pseudo laser scan.

$J_{c}\left(x,k\right)$ is represented by a one-sided quadratic barrier function. It uses the estimated drivable area, $d_{t}$, to enable the NMPC obstacle avoidance capability. For obstacle checking, a pseudo-laser scan was generated by taking the top view of the drivable area $d_{t}$. Subsequently, the contour of the drivable area is determined, and the distance from the camera to the drivable area contour is computed. This allows the generation of a vector of distances that resembles the output of 2D LiDAR (see Figure 4). The closest distance to the robot, $c_{d}\left(x,k\right)$, was used to generate a soft constraint that allowed collision-free motion, as presented in Equation (5).

(5) $$J_{c}\left(x,k\right)=\frac{1}{2}{\left(max\left\{0,c_{d}\left(x,k\right)\right\}\right)}^{2}.$$

The proposed MPM-based NMPC not only considers static obstacles such as poles and trees but also introduces a dynamic obstacle avoidance factor to evaluate the next motion to avoid collision in advance.

## 4. Experimental Results and Discussion

Intensive experiments were conducted in simulated and real-world environments to evaluate the feasibility of the proposed method. A simulation environment was built based on the Unreal Engine game editor. Training for the perception module was performed by generating sample images of a simulated robot equipped with a localization system and a front-facing color camera. The robot starts from an initial position and attempts to reach the goal destination while avoiding collisions with obstacles.

### 4.1. Perception Module Verification in Simulation

This sub-section briefly discusses the performance evaluation of semantic segmentation models used for drivable area estimation in a simulated environment. For semantic segmentation, different evaluation matrices can be used to demonstrate the performance of the proposed deep learning model [44]. The evaluation matrices used in this study were as follows:

#### 4.1.1. Pixel Accuracy ($P_{acc}$)

This is the most extensively used valuation benchmark for semantic segmentation []. It is defined as the accuracy of pixel-wise forecasting, where the comparison has been made in each pixel one-at-a-time with the ground truth mask, given as:

(6) $$\begin{array}{c} P_{acc}=\frac{\sum_{i=0}^{k}\left(P_{ii}\right)}{\sum_{i=0}^{k}\sum_{j=0}^{k}\left(P_{ij}\right)}, \end{array}$$

where *k* represents the total number of pixels in the test image, and $P_{ii}$ is predicted pixels as class *i*, and the ground truth is represented as $P_{ij}$, which is the number of pixels of class *i* predicted as class *j*.

#### 4.1.2. Intersection over Union (IoU)

The IoU metric, also known as the Jaccard index, is a commonly used evaluation metric for calculating the performance of segmentation models. It is generally used to calculate the performance by calculating the intersection and union between the ground truth and prediction, expressed as:

(7) $$\begin{array}{c} IoU=J_{a}\left(A,B\right)=|A\cap B|/|A\cup B|, \end{array}$$

where *B* shows the predicted segmentation maps, and *A* represents the ground truth.

#### 4.1.3. Mean-IoU (mIoU)

Another comprehensively used metric for semantic segmentation models is mean *IoU*. This was intended as the average value of IoU overall label classes. Substantially, they are used to outline the potential of semantic segmentation models. It usually ranges between 0 and 100 and is given as:

(8) $$\begin{array}{c} mIoU=\frac{1}{k+1}\sum_{i=0}^{k}\frac{t_{p}}{\sum_{j=0}^{k}f_{n}+\sum_{j=0}^{k}f_{p}-f_{n}}, \end{array}$$

where *k* represents the total classes, $t_{p}$ is the number of true positives, and $f_{p}$ and $f_{n}$ are false positives and false negatives, respectively.

**Figure 5:**
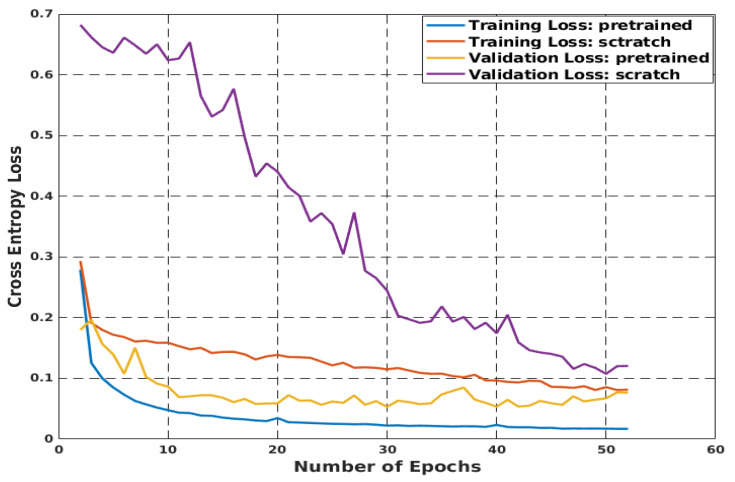
Comparison between model from scratch and model initialized with pre-trained classification weights.

**Figure 6:**
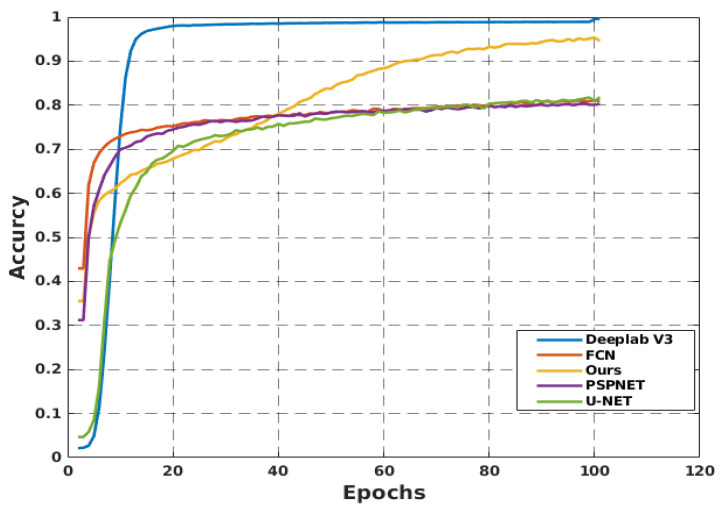
Comparison of the proposed method with baseline methods in terms of accuracy.

In this study, the segmentation model was trained on a synthetic dataset and tested in a real-world setting. First, the model was trained from the first layer without using pretrained weights, owing to the small size of the dataset. Subsequently, training the model from scratch precedes very strong overfitting, as shown in Figure 5, which shows that the training loss does not decrease. In the second test, the model was initiated using ImageNet classification pretrained weights. Figure 5 shows that the training loss converges smoothly without overfitting. The predicted binary semantic segmentation results for both training scenarios are shown in Figure 6. It is clear that transfer learning helps to increase the model accuracy outperforming the model trained from scratch, as the mIoUs shows.

**Figure 7:**
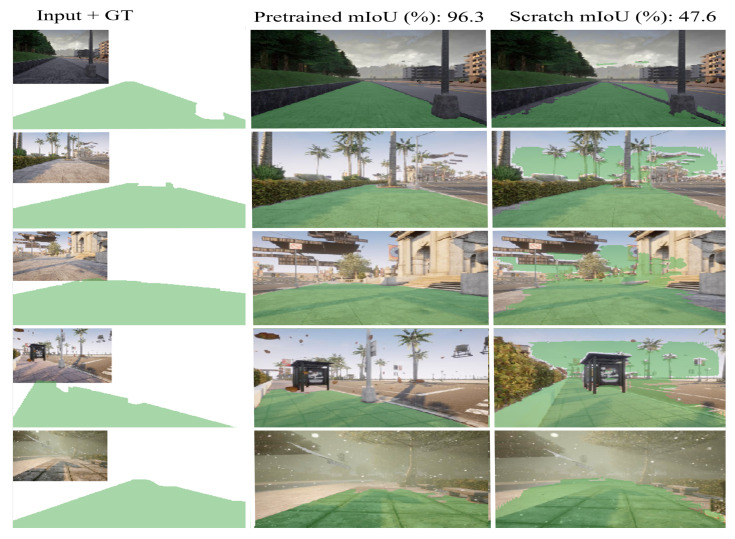
Binary semantic segmentation results from synthetic data in different simulation environment scenarios. The first column presents the image input and the corresponding ground truth (GT) semantic label. The second column shows the obtained results from the model that was pretrained. The third column presents the obtained results from the model that was trained from scratch.

A comparative study is conducted to demonstrate the effectiveness of the proposed segmentation model. DeeplabV3 [26], PSPNET [27], FCN [28], and U-Net [29] architectures were used as a baselines for the proposed model. Figure 7 shows that DeepLabV3 demonstrated preferable outcomes in the context of model accuracy. However, owing to the substantial number of parameters in DeepLabv3, it is not suitable for real-time operation on a single-board device.

**Figure 8:**
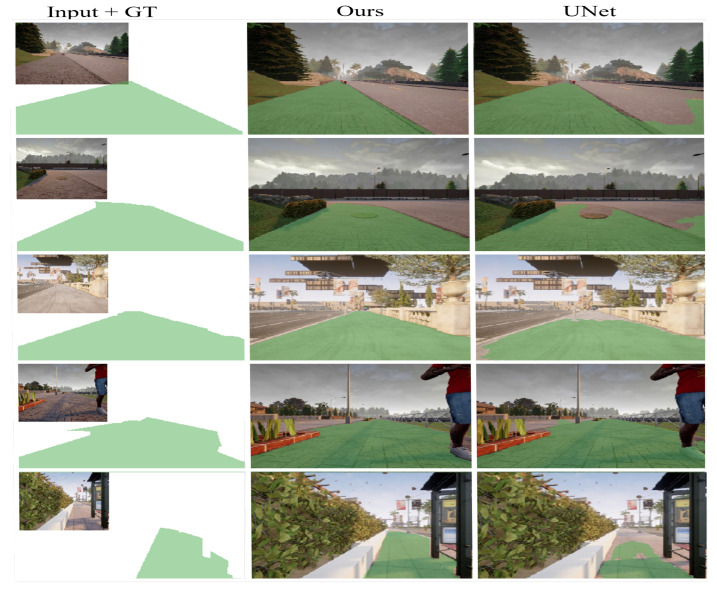
Semantic segmentation results of the proposed method and the U-Net model. The first column presents the image input and the corresponding ground truth (GT) semantic label. The second column presents the results obtained from our model. The third column presents the results obtained from the UNet model.

In contrast, our proposed method achieves acceptable performance, outperforming U-NET, FCN, and PSPNET with a trivial set of parameters, and manages to run in real-time as well. Table 2 presents a detailed comparison of the proposed scheme and baselines. DeepLabV3 shows high accuracy in terms of the PA, MPA, and mIoU. However, owing to their large parameter values, a large amount of computational power is required. By contrast, our model can be run using only a standard computer. The sample segmentation outputs from the proposed model and U-Net model are shown in Figure 8, where U-NET fails to segment the free space precisely. Distinctly, our model does not have false positives.

### 4.2. Motion Planning Module Verification in Simulation

**Figure 9:**
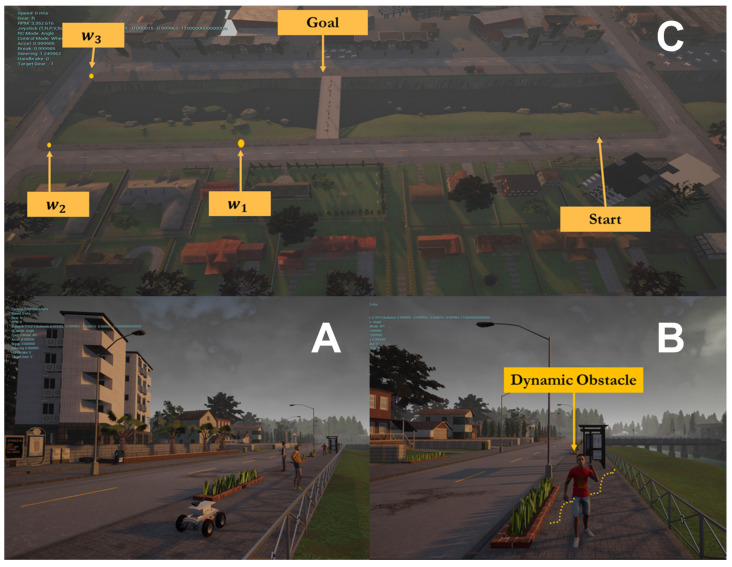
Simulation environment: (**A**) mobile robot with a forward facing camera. (**B**) Dynamic and static obstacles are included in the sidewalk. (**C**) Given a set of waypoints $w_{1},w_{2},w_{3}$, the robot must reach the goal point.

**Figure 10:**
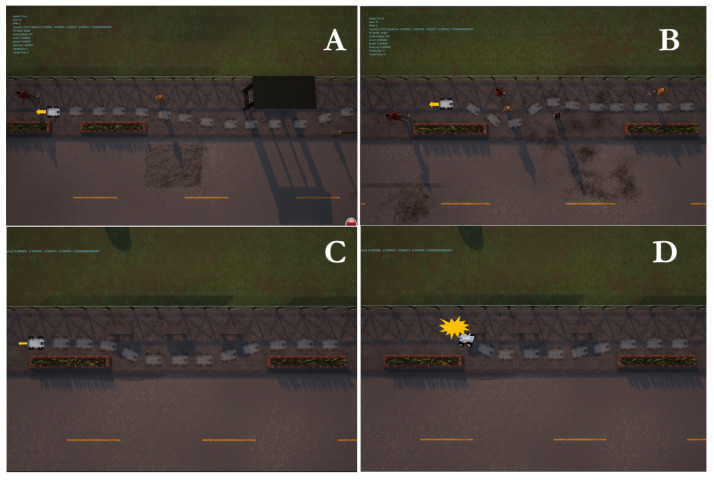
Different emerging behaviors during simulation experiments. At each snapshot, a long exposed trajectory is shown. (**A–C**) The robot adjusts its heading and forward velocity to avoid obstacles along the predicted trajectory using the proposed approach. (**D**) Snapshot shows where the robot crashed using the DWA algorithm.

**Figure 11:**
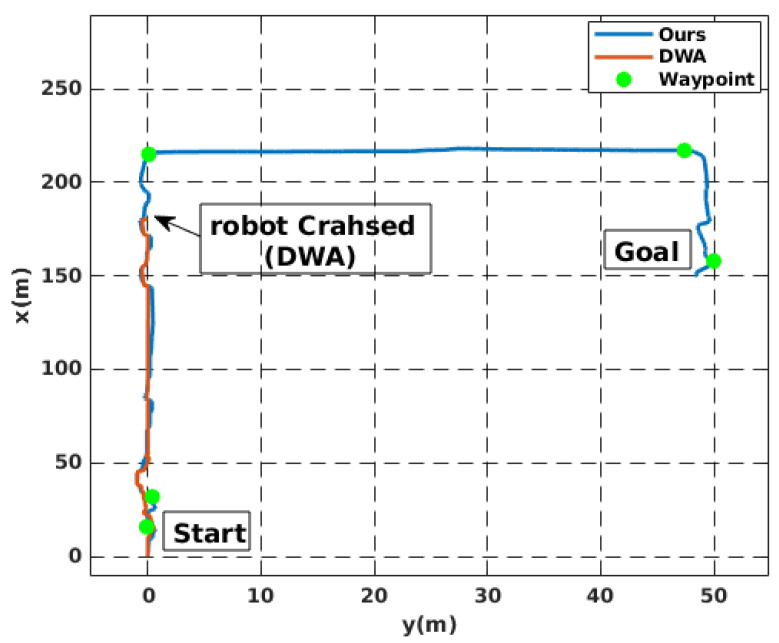
The path followed by the robot while avoiding obstacles in a simulated environment.

**Figure 12:**
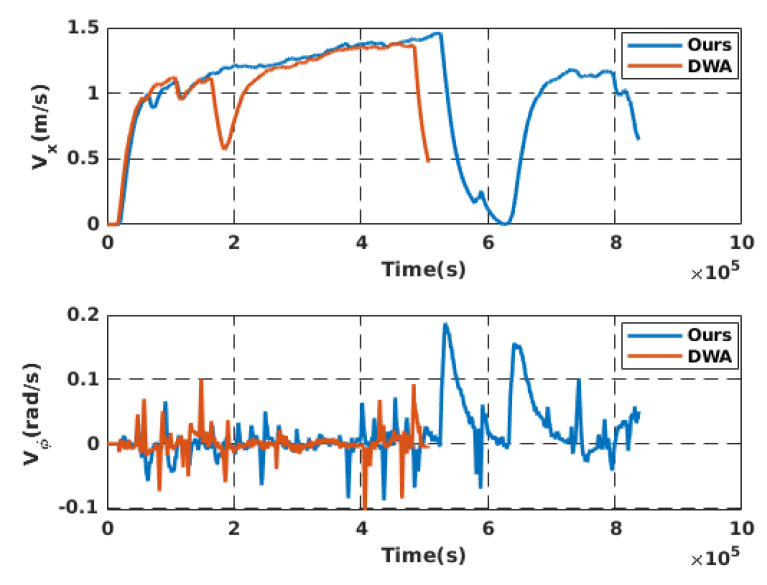
(**Top**): The commanded forward velocity in simulation. (**Bottom**): The commanded heading rate of change.

Simulation experiments were conducted to validate the proposed local motion planning module. First, a dynamic sidewalk environment was created using the Unreal Engine game editor, as shown in Figure 9. It contains animated walking pedestrians that act as dynamic obstacles and different static obstacles, such as bus stands, road borders, and public seats. The objective of the mission was to navigate the robot from the initial starting point to the destination. The proposed algorithm was benchmarked using the conventional dynamic window approach, DWA [45]. Our method demonstrates its potential while moving through a narrow sidewalk and more complex maneuvers, such as dynamic collision avoidance. However, the DWA showed a jerky motion, and the robot crashed before reaching the final goal, as depicted in Figure 10D. Moreover, the proposed approach automatically changes the robot’s heading and avoids obstacles and then returns to the sidewalk center once the obstacle is passed (see Figure 10 and the attached video). Figure 11 shows the path taken by the robot. The blue line represents the path while following the smooth commands, $v_{x},v_{\phi }$, generated by the proposed scheme. The commanded velocities are shown in Figure 12. The first row represents the linear forward velocity $v_{x}$. It can be observed that our algorithm generates smoother commands without violating the speed limit of 1.5 m/s. At the time stamp $t=6\times 10^{5}$, the speed was approximately 0 m/s. There was no available drivable area in this scenario owing to the walking person who took over the entire free space, and the robot speed increased when the person moved away. The second row presents the commanded angular velocities. Compared to the DWA command, our method shows a more stable velocity command while respecting the imposed constraints. It is recommended that the reader watch supplementary videos to better understand the simulated scenario.

### 4.3. Hardware Experiments

The hardware used for the real-time experimentation is Scout 2.0, developed for industrial applications by Agile Robotics, which has a compact body and low power consumption. In addition, scout 2.0 has its central control system, allowing us to customize complex modes of operation. Users can communicate with the central controller through the CAN Bus protocol, and an open-source SDK and robot operating system (ROS) package are provided.

**Figure 13:**
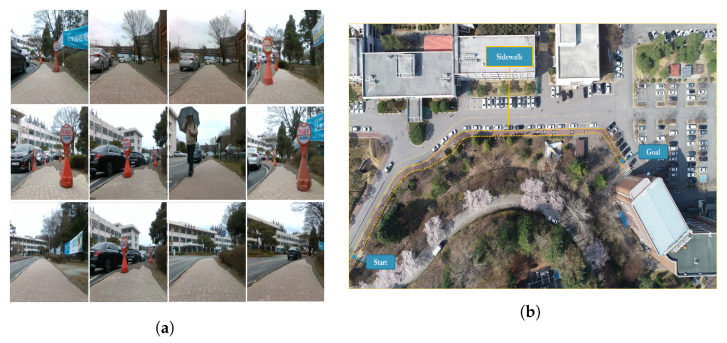
Zero-shot sim-to-real transfer: The segmentation model was deployed into a real robot. The robot performs autonomous navigation in real-world settings. (**a**) Examples of segmentation results on a real sidewalk. (**b**) Test site used for real-world experiments.

The optimized neural network and the presented controller were embedded into the robot PC. It comprises a CPU Intel Core i7-7567U processor @ 3.5 GHz Turbo and DDR4 RAM of 32 GB. With such configurations, the camera attached to the Scout provides RGB images at 30 FPS, while an optimized deep neural network can process the RGB images on the CPU at 22 FPS (sensor configuration for the real-time experimentation is shown Figure 1). Intensive real-world experiments have shown that the proposed approach performs comparably to the simulation results of a real mobile robot. Motions such as heading and forward speed adjustment and aligning to the sidewalk center were observed. The accompanying videos were available for real-time experiments. Figure 13b shows the environment in which the experiments were conducted. Figure 13a shows the real-time results of the semantic segmentation of the drivable area on a sidewalk. The method effectively translates from the simulation into the real world and steers clear of those objects that were not comprehended in the labeling class.

**Figure 14:**
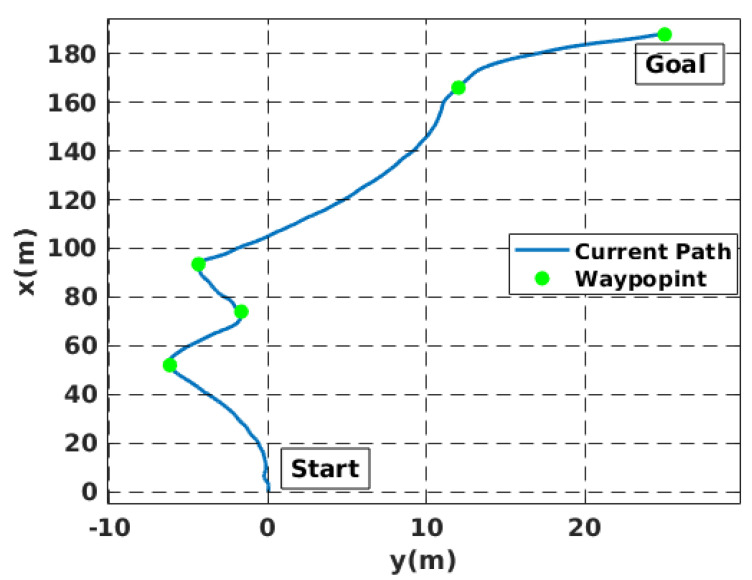
The followed path by the robot while avoiding obstacles in real-world settings.

**Figure 15:**
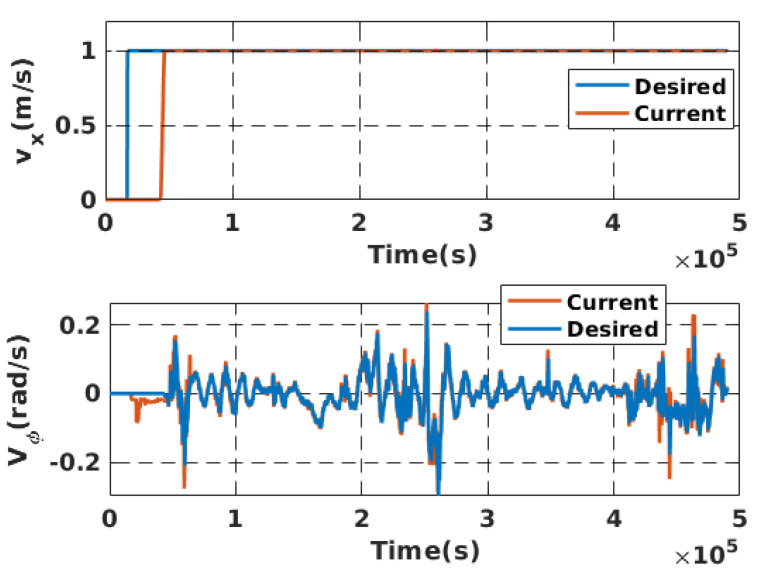
(**Top**): The commanded forward velocity. (**Bottom**): The commanded heading rate of change.

Figure 14 shows that the robot always reaches the desired waypoints successfully without crashing with the pedestrian and the road cones that were added to make the foot path more complex. Moreover, Figure 15 presents the evolution of two speeds (forward and angular) of the mobile robot. For example, the first row shows that the mobile robot tracked the desired speed without overshoot. As soon as obstacles or turns start to appear, the NMPC system provides larger angular velocities to change the robot heading, as the bottom row presents. Once the robot is oriented toward the waypoint and there is free space, the angular speed becomes approximately zero. The obtained results demonstrate the effectiveness of our approach in terms of obstacle avoidance and autonomous navigation.

## 5. Conclusions

In this paper, we presented a vision-based NMPC that enables a mobile delivery robot to autonomously navigate cluttered environments, deal with dynamic obstacles, and reach specified goal points to perform delivery missions. In contrast to the existing methods, the problem is formulated as an optimization problem that considers collision-free space and kinematic constraints over a specified preceding horizon. To achieve collision avoidance, a semantic segmentation deep learning model was created and trained from a synthetic dataset collected from a simulated dynamic sidewalk environment that was built using Unreal Engine software. The proposed segmentation model involves a transfer learning technique to reduce training time and increase sim-to-real transferability. The estimated drivable free space is used with a relaxed barrier function in the NMPC formulation without increasing the computational complexity of the problem. The delivery robot could automatically discover complex motions and avoid cluttered scenes with moving obstacles. In addition, real-world tests verified that the proposed algorithm can achieve zero-shot sim-to-real transfer. The proposed vision-based NMPC improves the safety and autonomy of the last-mile delivery robot.

## Figures and Tables

**Table 1:** The overall encoder architecture. *t* is the expansion factor, *c* is the number of output channels, *n* is the number of repeated layers and *s* is the stride.

Input	Operator	t	c	n	s
224${}^{2}$×3	conv2D	-	32	1	2
112${}^{2}$×32	bottleneck	1	16	1	1
112${}^{2}$×16	bottleneck	6	24	2	2
56${}^{2}$×24	bottleneck	6	32	3	2
14${}^{2}$×64	bottleneck	6	96	3	1
14${}^{2}$×96	bottleneck	6	160	3	2
7${}^{2}$×160	bottleneck	6	320	1	1
7${}^{2}$×320	conv2D 1×1	-	1280	1	1
7${}^{2}$×1280	avgpool 7×7	-	-	1	-
1${}^{2}$×1×1280	conv2D 1×1	-	k	-	60

**Table 2:** Comparison of the proposed scheme with the baseline methods in terms of parameters, pixel accuracy PA, MPA, and IoU.

Method	Parameters	PA%	MPA%	MIoU%
Deeplab-V3	41.25 M	97.46	93.84	96.05
PSPNET-MobileNet	63.967 M	84.91	72.53	77.03
FCN8-VGG	134.286 M	83.19	74.63	78.43
U-Net-RGB	31.033 M	76.31	68.54	78.50
Ours	3.984 M	94.41	91.59	93.78

## Data Availability

Not applicable.

## Supplementary Materials

The supplementary videos for the actual tests can be accessed at https://youtu.be/_DCzwDDRok0?list=PLF6aV0nw8UMyqvBiYEGdUR6tMVQ9oqMNF.

## Abbreviations

The following abbreviations are used in this manuscript:

NMPC	nonlinear model predictive control
SADR	sidewalk autonomous delivery robot
DWA	dynamic window approach
IMU	inertial measurement unit
RL	reinforcement Learning
CNN	convolution neural networks
